# Transfer Entropy between Communities in Complex Financial Networks

**DOI:** 10.3390/e21111124

**Published:** 2019-11-15

**Authors:** Jan Korbel, Xiongfei Jiang, Bo Zheng

**Affiliations:** 1 Section for Science of Complex Systems, Medical University of Vienna, Spitalgasse 23, 1090 Vienna, Austria; 2 Complexity Science Hub Vienna, Josefstädterstrasse 39, 1080 Vienna, Austria; 3 Faculty of Nuclear Sciences and Physical Engineering, Czech Technical University in Prague, Břehová 7, 115 19 Prague, Czech Republic; 4 College of Finance and Information, Ningbo University of Finance and Economics, Ningbo 315175, China; xfjiang@nbdhyu.edu.cn; 5 Department of Physics, Zhejiang University, Hangzhou 310027, China; zhengbo@zju.edu.cn; 6 School of Information Engineering, Ningbo Dahongying University, Ningbo 315175, China

**Keywords:** financial networks, community structure, information transfer, Rényi entropy

## Abstract

In this paper, we analyze information flows between communities of financial markets, represented as complex networks. Each community, typically corresponding to a business sector, represents a significant part of the financial market and the detection of interactions between communities is crucial in the analysis of risk spreading in the financial markets. We show that the transfer entropy provides a coherent description of information flows in and between communities, also capturing non-linear interactions. Particularly, we focus on information transfer of rare events—typically large drops which can spread in the network. These events can be analyzed by Rényi transfer entropy, which enables to accentuate particular types of events. We analyze transfer entropies between communities of the five largest financial markets and compare the information flows with the correlation network of each market. From the transfer entropy picture, we can also identify the non-linear interactions, which are typical in the case of extreme events. The strongest flows can be typically observed between specific types of business sectors—financial sectors is the most significant example.

## 1. Introduction

Complex networks are systems exhibiting a broad range of non-trivial emergent phenomena, including extreme shocks, long-term memory and power-law dynamics [1,2,3], long-range correlations [4,5,6,7] or multifractality [8,9,10,11]. The collective behavior can be modeled by many approaches, complex dynamical models [12], agent-based models [13,14] or graph theory [15,16,17], to name just a few. One of the most important and most difficult tasks is to describe the collective behavior of interacting edges, and corresponding information flows between them.

There exist several techniques for the measurement of the information flows. To the most popular methods belong lagged cross-correlation [7] or Granger causality [18,19]. Unfortunately, these quantities suffer from some shortcomings. The main disadvantage of cross-correlation is the fact that it is not possible to separate effects caused solely by the source time series and effects of the environment, e.g., the effects of common information sources. For Granger causality, it is possible to detect information coming only from the source series. Nevertheless, both measures are based on linear models and can be therefore insensitive to nonlinear interactions.

These issues can be overcome by the introduction of information-based measures that can appropriately detect the information flows and identify its sources. Transfer entropy, introduced by Schreiber [20], is the model-free measure of information transfer between time series. It is based on famous Shannon information entropy and has been successfully used in many applications [21,22,23,24,25]. It has been shown that Granger causality and transfer entropy coincide in the “Gaussian world” [26]. Nevertheless, most complex networks are highly non-Gaussian and nonlinear. With the advent of generalized entropies, many applications of entropy in thermodynamics, statistics and information theory found their natural generalizations. These generalizations can be utilized to describe complex and nonlinear dynamics more precisely. Following this scheme, Jizba et al. introduced a new class of Rényi transfer entropies [27]. The specifics of Rényi transfer entropy is given by the fact that it is possible to focus on information transfer of certain parts of probability distributions. Since then, Rényi transfer entropy has found several applications, e.g., in signal processing [28] or single-spin dynamics [29]. The topic of information transfer remains a hot topic of ongoing research [30,31,32].

The main aim of this paper is to investigate methods for the detection of information flows between communities in complex networks. As an example, we demonstrate the method on information flows between communities in financial markets. Financial markets can be treated as complex networks with the inner structure of communities [33,34,35,36]. Typically, these communities correspond to business sectors [37,38]. So far, information flows have been measured only between financial markets [21,22,27] or between single stocks in one particular market. Our aim is not only to detect particular information flows but more generally to understand how specific types of business sectors interact with each other. Such nonlinear interactions become even more prominent during some unusual trading period, such as, e.g., financial crises, external driving, or company affairs. Distribution of marginal events can be accentuated by choosing a proper *q* for the Rényi transfer entropy. All these aspects can help us understand the different dynamics of particular markets.

The rest of the paper is organized as follows: Section 2 discusses the properties of the correlation function and its spectral decomposition into different modes. Section 3 describes the algorithm for the detection of the community structure in complex networks. Section 4 introduces the concept of transfer entropy and discusses its generalization based on Rényi entropy. Section 5 presents the structure of information transfer within and between communities in financial markets and discusses the differences between particular markets. Moreover, it compares flows of rare events by Rényi transfer entropy. The last section is dedicated to conclusions.

## 2. Correlation Function and Sector Correlation

Correlation is one of the most important measures detecting the similarity of time series. For a pair of stationary time series X(t), Y(t), it is possible to introduce a cross-correlation function as

(1) $$C_{X,Y}\left(\tau \right)=\frac{\langle \left(X\left(t\right)-\mu_{X}\right)\left(Y\left(t-\tau \right)-\mu_{Y}\right)\rangle }{\sigma_{X}\sigma_{Y}},$$

where $\mu_{X}$ and $\mu_{Y}$ are mean values of each time series and $\sigma_{X}$, $\sigma_{Y}$ are their standard deviations. Naturally, C∈[−1,1]. We can distinguish three different cases. First, for X=Y we obtain an auto-correlation function. It is used to detect dependencies in the time series.

Second, for τ=0 we get equal time cross-correlation, which can be treated as a measure of similarity between two series. It cannot be used as the measure of information flow because it lacks directionality and can be affected by the effects of the environment. Nevertheless, it is used in many standard techniques, including the detection of community structure, as shown in Section 3.

Finally, for X≠Y and τ>0, we get lagged cross-correlation. It has clear directional meaning. Unfortunately, it is hard to distinguish between causality and other forms of dependence, and it may not be sensitive to nonlinear interactions. In the case of a noisy system like financial markets, cross-correlations decay within minutes, and it is not possible to detect the interactions beyond this scale in most cases. These issues serve as a motivation for the introduction of measures based on information theory, such as, e.g., mutual information or transfer entropy. We present the main results of information theory in Section 4.

### Mode Decomposition of Correlation Matrix

In a noisy environment with external information sources, the correlation matrix contains not only the information about interactions between the time series, but also global market movement and noise fluctuations. Let us define the correlation matrix C between time series $X_{i}\left(t\right)$ and $X_{j}\left(t\right)$ as

(2) $$C_{ij}=C_{X_{i},X_{j}}\left(\tau =0\right)\;=\langle X_{i}-\langle X_{i}\rangle .$$

The spectrum of the correlation matrix C is real because of the symmetry. The matrix C can be represented via its spectral decomposition

(3) $$C=\sum_{\alpha }\lambda_{\alpha }u_{\alpha }⊗u_{\alpha },$$

where $\lambda_{\alpha }$ are the eigenvalues, and $u_{\alpha }$ are the eigenvectors. The correlation matrix for *N* uncorrelated time series in finite time *T* is known as Wishart matrix and the distribution of its eigenvalues is for N→∞, T→∞, Q=T/N≫1 given by [39,40]

(4) $$P\left(\lambda \right)=\frac{Q}{2\pi }\frac{\sqrt{\left(\lambda_{max}-\lambda \right)\left(\lambda -\lambda_{min}\right)}}{\lambda },$$

where $\lambda_{max/min}={\left[1\pm {\left(1/Q\right)}^{1/2}\right]}^{2}$. Thus, the eigenvalues not belonging to this range represent the non-random interactions of the system. Let us order the eigenvalues, so $\lambda_{i}\geq \lambda_{i+1}$. In many systems, like financial markets, the largest eigenvalue represents the overall system (market) mode [41]. Local interactions can be described by the sector correlation $C^{sec}$ which is defined as

(5) $$C^{sec}=\sum_{\alpha =2}^{\alpha_{max}}\lambda_{\alpha }u_{\alpha }⊗u_{\alpha },$$

where $\lambda_{\alpha_{max}}$ is the smallest eigenvalue larger than $\lambda_{max}$.

## 3. Community Structure in Complex Networks

The correlation matrix (or sector correlation matrix) contains the full information about all interactions of the network constituted by particular stocks. Unfortunately, the number of links is N2, which becomes a huge number for large networks, while only a small fraction of links play a relevant role in the dynamics of the network. Therefore, it is not only desirable but also necessary to discard most of the links and keep the most significant ones. To this end, several simple algorithms have been proposed. The minimal spanning tree (MST) algorithm [42] is based on a simple idea. Having correlations ordered in the descending order, we add the link to the filtered graph. If the addition of the edge would create the circle, we do not add it and move to the next edge, until we obtain the fully connected graph. Such a graph contains *N* nodes and N−1 edges and maximizes the aggregated correlation of the graph.

This simple algorithm has nevertheless a distinctive flaw. Because of the tree structure, significant correlations that would create a circle are omitted. Such circles can describe a small set of stocks strongly interacting with each other. Therefore, a generalization of the MST method was proposed in Ref. [43]. The method is called Planar maximally filtered graph (PMFG). It is based on a similar idea as the MST algorithm, but the condition of no graph circles is replaced by the planarity condition, i.e., that it is possible to embed the graph in a plane. The generated graph can be viewed as a triangulation of the plane. It is possible to show that the PMFG graph consists of 3N−6 edges.

Complex networks usually consist of nontrivial structures of highly connected nodes, which create communities. Interactions in the communities are typically very strong, while interactions between communities vary from relatively strong to very weak. The importance of community detection was pointed out by many authors [33,34]. One of the successful methods which is used for community detection is called InfoMap. It has been introduced in Refs. [35,36] and is based on the optimal compression of information flows in the networks. The main idea is to minimize the average code describing the random walk of the network. Typically, the walker remains in one community for a long time and then suddenly jumps to another community. In financial markets, the approach was successfully used, together with the correlation decomposition method, in Ref. [44] to reveal the structure of business sectors.

## 4. Transfer Entropy

The concept of entropy was introduced by Shannon. According to Campbell coding theorem [45], it represents the minimal amount of binary information necessary to encode a message. It can be expressed as

(6) $$H\left(X\right)=-\sum_{x}p\left(x\right)\log_{2}p\left(x\right),$$

where p(x) is the probability distribution of occurrence of symbols from the alphabet ${\left\{A_{i}\right\}}_{i=1}^{S}$. We denote the discrete random variable as *X*. Note that the information entropy is closely related to well-known thermodynamic Boltzmann-Gibbs entropy via the multiplicative factor $k_{B}$ (Boltzmann constant). It is important to say that the term H(X) represents only information contained in *X*. Analogously, information contained in X∪Y can be represented by the joint entropy H(X∪Y) given by the joint distribution p(x,y). If *X* is statistically independent of *Y*, the joint entropy is just H(X∪Y)=H(X)+H(Y). Conditional entropy can be introduced as

(7) $$\begin{array}{c} H(Y|X)=H(X\cup Y)-H(X). \end{array}$$

This allows us to introduce mutual information of *X* and *Y* as

(8) $$\begin{array}{c} I(X;Y)=H(X)-H(X|Y)=H(Y)-H(Y|X)\\ =H(X)+H(Y)-H(X\cup Y). \end{array}$$

Information flow from time series Y(t) to time series X(t) can be introduced as lagged mutual information I(X(t);Y(t−τ)). Nevertheless, although directional, it also includes mutual information, which is induced by a common external source, e.g., when both *X* and *Y* are statistically dependent on a common random variable *Z*. In this case, it is convenient to express the mutual information conditioned by the source *Z* by

(9) I(X;Y|Z)=H(X|Z)−H(X|Y∪Z).

Let us consider discrete time series X(t), Y(t) with a constant time lag τ. Let us denote $x_{m+1}=X\left(m+1\right)$, $X_{m}=\left\{X\left(m\right),\cdots ,X\left(1\right)\right\}$ and $Y_{m}^{l}=\left\{Y\left(m\right),\cdots ,Y\left(m-l+1\right)\right\}$. For stationary series is $p\left(X_{m}\right)∼p\left(X_{m+t}^{m}\right)$, and similarly $p\left(Y_{m}^{l}\right)∼p\left(Y_{m+t}^{l}\right)$. Shannon transfer entropy (STE) is defined as the conditional mutual information

(10) $$T_{Y\to X}\left(m,l\right)=I\left(x_{m+1};Y_{m}^{l}|X_{m}\right)\;.$$

The definition is clearly directional. Moreover, it takes into account only the dependency whose origin is in source series *Y*. Dependencies caused by common external sources are not taken into account. It is possible to write it explicitly as

(11) $$\begin{array}{c} T_{Y\to X}\left(m,l\right)=-\sum p\left(x_{m+1},X_{m},Y_{m}^{l}\right)\log_{2}\frac{p(x_{m+1}|X_{m})}{p(x_{m+1}|X_{m},Y_{m}^{l})}\;. \end{array}$$

Naturally, transfer entropy depends on history indices *m* and *l*. For Markov processes of order *m* and *l* it is sufficient to use the history up to the order of the processes. Unfortunately, for non-Markovian processes, i.e., processes with a long history, this is not possible. Ideally, one should take into account the whole history of the series, i.e., m→∞ to find a stable value independent of *m*. Unfortunately, this is not possible due to the finite size of the dataset. This might be an issue even for very long datasets because the number of possible states grows as $S^{m+l}$, which also affects computational time and accuracy. Therefore, Marchinski and Kantz introduced the effective transfer entropy [21] to avoid the problems with finite-size effects and with spurious information transfer as

(12) $$T_{Y\to X}^{eff}\left(m,l\right)=T_{Y\to X}\left(m,l\right)-T_{Y_{\mathrm{shuffled}}\to X}\left(m,l\right)\;.$$

The typical choice of parameters is then m=1 and l=1 [21].

### Rényi Transfer Entropy

Shannon entropy is not the only one functionally fulfilling the Khichin additivity axiom for conditional entropy. It has been shown by Rényi [46] that there exists the whole class of entropies, characterized by parameter q>0, which can be expressed as

(13) $$S_{q}\left(X\right)=\frac{1}{1-q}\log_{2}\sum_{x}p{\left(x\right)}^{q}\;.$$

In the limit q→1, we recover the Shannon entropy. One can formulate (similarly to Shannon entropy) the coding theorem for Rényi entropy. It describes the minimal cost necessary to encode a message when the cost is an exponential function of the message length [47]. Rényi entropy is closely related to multifractal systems [46,48] and escort distributions, which can be defined as

(14) $$\rho_{q}\left(x\right)=\frac{p{\left(x\right)}^{q}}{\sum_{x^{\prime }}p{\left(x^{\prime }\right)}^{q}}\;.$$

This class of distributions conforming a group was originally introduced in connection with chaotic systems [49]. They are also called “zooming distributions”, because for q<1, they highlight tail parts of the distribution, while for q>1, they suppress them and accentuate central parts of the distribution.

Contrary to other generalized entropies, the relevant operational definitions of mutual information (Equation (8)) and conditional mutual information ((Equation (9)) remain Rényi entropy valid, which is the consequence of additivity. Consequently, Rényi transfer entropy (RTE) can be expressed as

(15) $$\begin{array}{c} T_{q;Y\to X}\left(m,l\right)=\frac{1}{1-q}\log_{2}\frac{\sum \rho_{q}\left(X_{m}\right)\;p^{q}\left(x_{m+1}|X_{m}\right)}{\sum \rho_{q}\left(x_{m+1},X_{m},Y_{m}^{l}\right)\;p^{q}\left(x_{m+1}|X_{m},Y_{m}^{l}\right)} \end{array}$$

which for q→1 again boils down to STE.

The main difference between STE and RTE is that we can focus on different parts of distributions by varying the parameter *q*. Contrary to STE, RTE can be negative, which is equivalent to the situation when

(16) $$S_{q}\left(x_{m+1}|X_{m}\cup Y_{m}^{l}\right)\geq S_{q}\left(x_{m+1}|X_{m}\right)\;.$$

This paradoxical behavior brings desirable information that the knowledge of historical values *X* and *Y* reveals an extra risk to certain parts of distribution coming from nonlinear interaction between stocks. Consequently, RTE cannot be interpreted as the strength of information. On the other hand, it is possible to understand the negative RTE as the presence of emergent collective interactions among the stocks, leading to the increased complexity of the network.

We are typically interested in information transfer of the tail parts of the distribution, i.e., swan-like events. Naturally, these events do not occur very often, but they can remarkably affect the whole network. These events can spread in the network and cause an avalanche effect. For the detection of swan-like events, it is possible to adjust the parameter *q* to values smaller than one. Because for small values is the method very sensitive to errors, one usually chooses a compromise value, e.g., q=0.75.

## 5. Information Transfer between Business Sectors in Financial Markets

In order to give an example of a system with complex interactions, let us turn our attention to the analysis of information transfer between business sectors in financial markets. We investigate five largest stock exchanges (SE) according to the market capitalization—New York SE, London SE, Tokyo SE, Shanghai SE, and Hong Kong SE. These markets have a sufficiently rich structure and contain stocks from various business sectors. Each market is represented by a set of assets included in one of the main market indices. For each market, we investigate the period of the last 10–16 years (ending in 2016). We include only stocks that have been traded at least 1000 days (approx. 4 years). Basic statistics of all markets, including the number of investigated stocks and average lengths of the time series is contained in Table 1. Each stock is represented by its price $S_{i}\left(t\right)$. Daily returns are given as

(17) $$r_{i}\left(t\right)=\ln S_{i}\left(t\right)-\ln S_{i}\left(t-1\right).$$

For each market, we calculate the correlation matrix of daily returns and extract the sector mode correlation from the spectral decomposition. The sector mode correlation can be used for the definition of the adjacency matrix of the financial market network, which is usually defined as $A_{ij}=1-C_{ij}$. We filter the edges with the help of the PMFG method and keep only edges with the lowest distance (largest correlation).

Using the InfoMap algorithm, we determine the communities of each market. These communities correspond very well to business sectors. Nevertheless, for some markets, it is possible to find communities consisting of two anti-correlated subsectors [37]. This evidence is confirmed in all investigated markets. The only exception is provided by large conglomerates operating in several business sectors. In some cases, like for Hong Kong SE, the business sectors are also influenced by the country of residence. The main sectors consist of eight types of companies: basic materials, consumer goods, financial services, industry, services, technology, healthcare, and utilities. The communities often correspond to a specific subsector/industry. A list of all industries and their abbreviations can be found in Appendix A. Additionally, the method identifies flows between communities according to their mutual correlations.

For each market, we calculate average transfer entropy between communities. In all calculations, we use the 3-letter alphabet with division the three-letter histogram (5%,90%,5%), i.e., five percent of the largest drops, ninety percent of the most common events and five percent of the largest rises. This division enables us to focus on the large market movements, and filter out the noisy background of the system. Similarly to the correlation network, we obtain the full directed network of information flows between communities. Again, only a fraction of these flows is significant. To this end, we use the bootstrap methods [50] and compare the flows with the surrogated data flows, which are the consequence of the finite-size effects. We take into account only flows, which significantly outperform the random effects obtained by the surrogated data. The threshold is chosen such that the significant flows are at least twice as large as the mean transfer entropy of the surrogated data.

Before analyzing each market separately, let us note several general remarks. In all cases, the correlation network is remarkably different from the information flow network for all markets. While community structure corresponds to the business structure determined by frequent interactions on the market, information flows reveal the structure hidden under regular interactions. Generally, information flows are strongest in two situations: first, between financial communities (e.g., banks, investment institutions or real estates) and second, large enterprises belonging to the key industry sectors of the particular country (car manufacturers in Germany or steel production industry in China). To be more specific, let us turn our attention to individual markets.

### 5.1. New York SE

**Figure 1:**
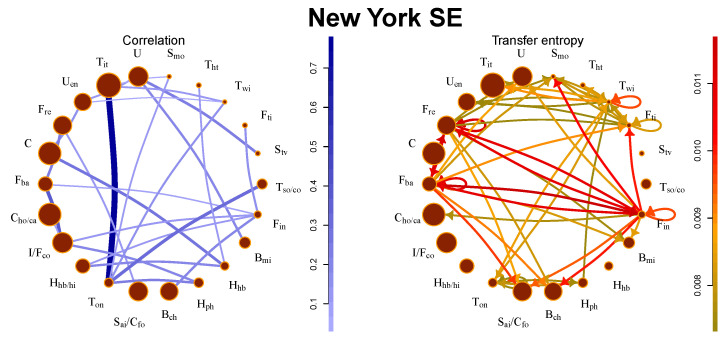
Comparison of correlation network and transfer entropy flows between communities of New York stock exchange (SE). The size of the circle denotes the number of stocks in the community, the strength of the line/arrow denotes the strength of the interaction.

This market exhibits the largest information flows among all investigated markets. We observe the strongest flows between financial communities. They also affect other communities (movie production, consumption, etc.). This is not a surprising fact because the real estate sector is in the U.S. tightly connected with the banking and investment sector and the financial crisis 2008 started in the real estate sector and spread to the banking sector. These connections are also present in the correlation picture, but the strongest correlations are observed in the technology industry. The comparison between the correlation network and transfer entropy flows is depicted in Figure 1. The stocks traded at New York SE exhibit the strongest correlations, largest information flows, and most significant complexity (as discussed in Section 5.6).

### 5.2. London SE

**Figure 2:**
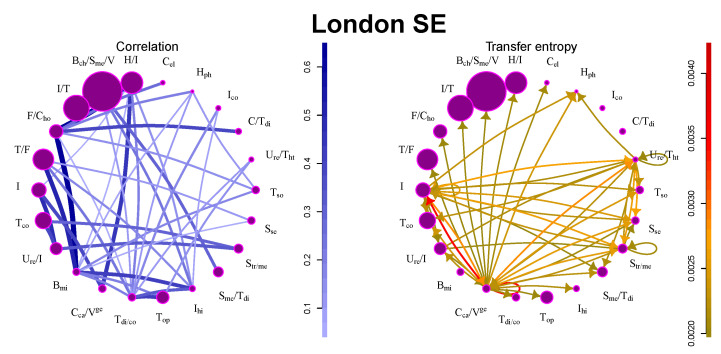
Information flows between communities for London SE. The size of the circle denotes the number of stocks in the community, the strength of the line/arrow denotes the strength of the interaction.

Contrary to New York SE, interactions at London SE are much weaker. Stocks are very weakly correlated, and there are not many large information flows (see Table 1 and Figure 2). The reason for this may be found in the structure of the market: most companies are industrial and technology companies producing various types of high-tech products or large multi-sector conglomerates. Their performance is not influenced much by the other companies in the market. The only community with significant outflows contains major German financial and service companies (Deutsche Bank, Frankfurt, Germany; Deutsche Post, Bonn, Germany; Allianz, Munich, Germany; Deutsche Börse, Frankfurt, Germany; Lufthansa, Cologne, Germany) and large German car manufacturers (BMW, Munich, Germany; Daimler Chrysler, Auburn Hills, MI, USA; Volkswagen, Berlin, Germany).

### 5.3. Tokyo SE

**Figure 3:**
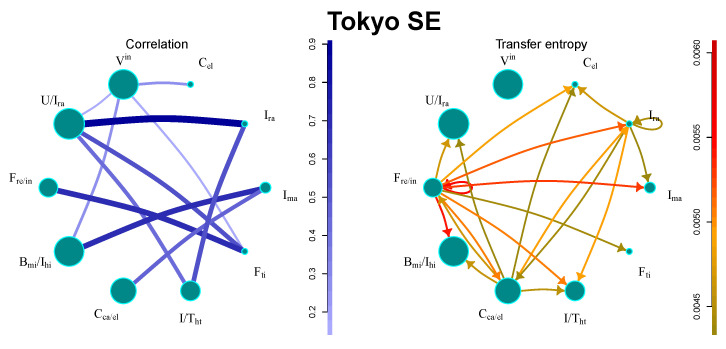
Information flows between communities for Tokyo SE. The size of the circle denotes the number of stocks in the community, the strength of the line/arrow denotes the strength of the interaction.

The market has a relatively small number of large communities, as presented in Figure 3. The strongest flows can be found between financial sectors. There are also industry sectors with significant flows, such as, e.g., electronics productions, or railway construction. Interestingly, the sector containing all international companies remains isolated.

### 5.4. Shanghai SE

**Figure 4:**
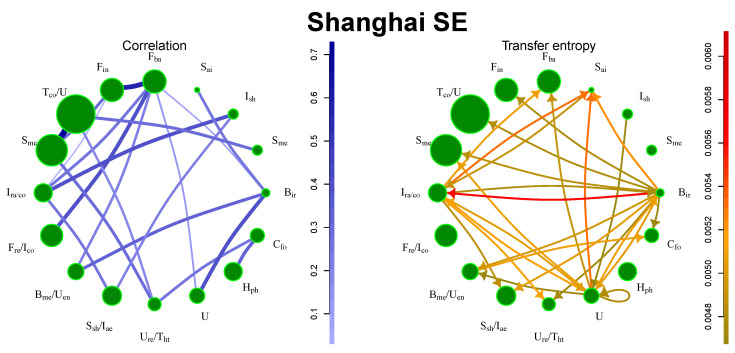
Information flows between communities for Shanghai SE. The size of the circle denotes amount of stocks in the community, the strength of the line/arrow denotes strength of the interaction.

It is possible to identify two sources of information, namely, the railway construction industry and steel production, as depicted in Figure 4. China is the largest steel producer and exporter and the sector has a great impact on the other areas of the Chinese industry. On the other hand, financial companies do not produce the strongest flows, because the financial companies are also listed in Hong Kong SE.

### 5.5. Hong Kong SE

**Figure 5:**
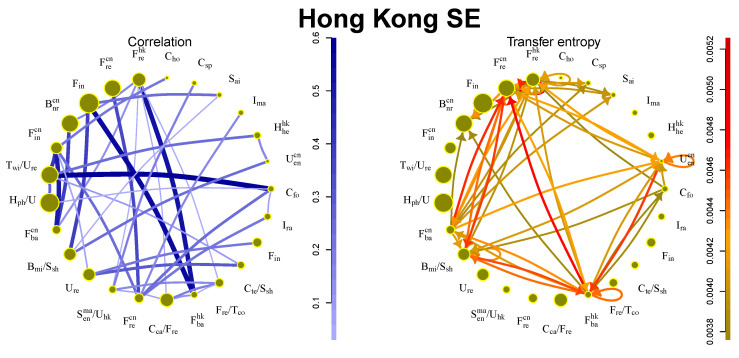
Information flows between communities for Hong Kong SE. The size of the circle denotes amount of stocks in the community, the strength of the line/arrow denotes strength of the interaction.

Interactions on the Hong Kong SE are influenced not only by affiliation to business sectors but also by the country of origin, as shown in Figure 5. Companies from mainland China and Hong Kong are contained in approximately the same amount. Since the Hong Kong market includes the large financial sector from both mainland China and Hong Kong, it is not surprising that the strongest flows are among financial sectors. The flows are strong also among sectors with a different country of origin.

### 5.6. Information Transfer of Rare Events and Market Complexity

**Figure 6:**
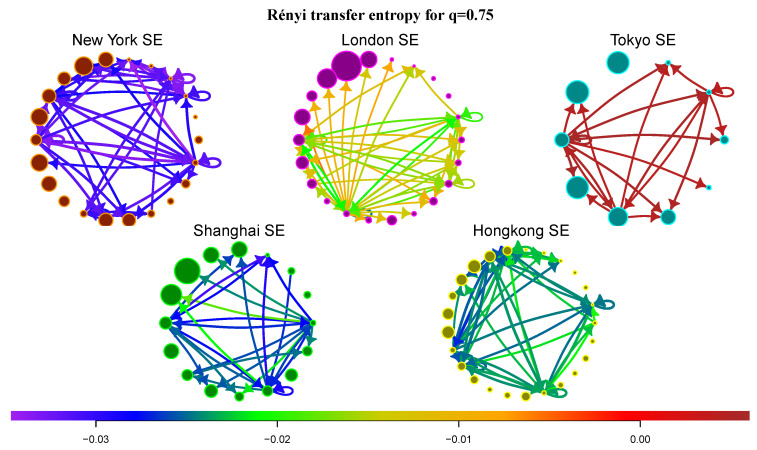
Rényi transfer entropy for the five financial markets.

Let us focus on RTE and the transfer of rare events. As discussed in Section 4, RTE for q<1 accentuates transfer of marginal events. We calculate the average RTE between communities for all markets and focus on the most significant flows, i.e., flows with the largest STE (see Figure 6). In Ref. [27] the authors analyzed Rényi information flows between indices of different financial markets. In most cases is RTE positive. The only exception is the information transfer between indices S&P 500, DJIA and NYSE 100. All these indices are created from stocks of New York SE, which points to the fact that interactions in New York SE are very sensitive to marginal events, which is also confirmed by our analysis. New York SE exhibits the lowest values of RTE for q=0.75 among all markets (average values of STE and RTE are listed in Table 1). This reflects the fact that New York SE is a well-developed market with a complex structure that recently passed through the large financial crisis (the crisis and post-crisis data constitute the major part of the investigated period). In contrast, London SE and especially Tokyo SE have much higher values of RTE, for some flows in London SE and all flows in Tokyo SE the RTE is even positive. Shanghai SE and Hong Kong SE are somewhere between these two types of behavior. Generally, the information transfer of swan-like events between markets is much more predictable than within financial markets, especially for New York SE.

## 6. Conclusions and Perspectives

The main aim of this paper was to investigate information flows between communities of complex networks. Information flows can be measured by transfer entropy, a model-free measure quantifying the amount of information transmitted from the source time series to the target time series. It can successfully describe complex systems with nonlinear interactions. As an example, we have analyzed the five largest financial markets. We find that the strongest flows are observed for financial sectors and key industry sectors (e.g., German car manufacturers at London SE or steel producers at Shanghai SE). On the other hand, sectors with high correlations, as technology sectors or consumer goods, exhibit much weaker information flows. This is caused by the fact that the former sectors produce significant information transfer of marginal events, which becomes much more important in the transfer entropy picture.

To emphasize the importance of rare events transfer, we introduced the Rényi transfer entropy which enables one to study information flows between specific parts of probability distributions. Rényi transfer entropy can acquire negative values, which can be interpreted as an additional risk (or uncertainty) for specific parts of the distribution of the target series. Negative Rényi transfer entropy can be interpreted as the increased complexity of the network. We have compared Rényi transfer entropy for q=0.75 among the five example markets. As a result, some markets, especially New York SE, exhibit negative Rényi transfer entropy for most flows, which signals that the transfer of rare events is nonlinear and less predictable—the network is complex. This should be taken into account when designing models of risk spread and in the modeling of swan-like events.

Dynamics of information flows measured by transfer entropy provides a different description of complex financial networks, when compared with interactions measured by correlations. Therefore, complex networks based solely on information transfer would provide a novel approach to the understanding of complex network dynamics. Because information flows are directional, it will be necessary to adjust the procedures to be able to deal with directed graphs. Investigation of communities based on directed transfer entropy-based networks is the subject of ongoing research.

## Figures and Tables

**Table 1:** Statistical properties of the investigated markets. For each market, the table contains number of investigated stocks, average length of trading period, equal-time cross-correlation, lagged cross-correlation, Shannon and Rényi transfer entropy and number of communities obtained from the InfoMap algorithm.

Market	New York	London	Tokyo	Shanghai	Hong Kong
Index	S&P 500	FTSE AS	NKY 225	SSE 300	HSI Comp.
stocks	485	527	185	283	411
av. length (days)	3905	2206	2205	2929	3677
$\bar{C}\left(0\right)$	0.2122	0.0390	0.2185	0.2601	0.1448
$\bar{C}\left(1\right)$	−0.0093	0.0069	0.0002	0.0193	0.0229
$\bar{STE}$	0.0047	0.0008	0.0027	0.0030	0.0019
${\bar{RTE}}_{0.75}$	−0.0260	−0.0062	0.0027	−0.0218	−0.0162
communities	22	22	10	16	25

## Appendix A

**Table A1:** Sectors and Industries.

**B**	**Basic Materials**	**C**	**Consumer Goods**	**F**	**Financial Services**
$B_{ch}$	chemistry	$C_{ca}$	car manufacture	$F_{ba}$	banks
$B_{ir}$	iron & steel	$C_{el}$	Electronics	$F_{co}$	consulting services
$B_{me}$	metals	$C_{fo}$	food	$F_{hi}$	health insurance
$B_{mi}$	mining	$C_{sp}$	sport & lifestyle	$F_{in}$	investment services
$B_{nr}$	natural resources	$C_{te}$	textile	$F_{re}$	real estate
		$C_{ho}$	household	$F_{ti}$	travel & accident insurance
**I**	**Industrial goods**	**S**	**Services**	**T**	**Technology**
$I_{ae}$	aerospace & defense	$S_{ai}$	airlines	$T_{co}$	communications
$I_{ag}$	agriculture industry	$S_{en}$	entertainment	$T_{di}$	digital services
$I_{hi}$	heavy industry	$S_{me}$	media	$T_{ht}$	high-tech industry
$I_{in}$	infrastructure	$S_{mo}$	movie production	$T_{it}$	information technology
$I_{ma}$	machinery	$S_{se}$	security services	$T_{on}$	online services
$I_{ra}$	railway construction	$S_{sh}$	shipping	$T_{op}$	optical & nano technology
$I_{sh}$	ship construction	$S_{tr}$	transportation	$T_{so}$	software
${}_{Ive}$	vehicle industry	$S_{tv}$	television	$T_{wi}$	wireless services
**H**	**Healthcare**	**U**	**Utilities**		**Country**
$H_{hb}$	health & beauty	$U_{en}$	energy	${}^{cn}$	China
$H_{me}$	medical equipment	$U_{ga}$	gas & oil	${}^{hk}$	Hongkong
$H_{ph}$	pharmacy	$U_{re}$	renewable energy	${}^{ma}$	Macau
				${}^{in}$	international
		**V**	**Various and conglomerates**	${}^{ge}$	major German companies
